# Effect of sodium bicarbonate supplementation on the renin-angiotensin system in patients with chronic kidney disease and acidosis: a randomized clinical trial

**DOI:** 10.1007/s40620-020-00944-5

**Published:** 2020-12-31

**Authors:** Dominique M. Bovée, Lodi C. W. Roksnoer, Cornelis van Kooten, Joris I. Rotmans, Liffert Vogt, Martin H. de Borst, Robert Zietse, A. H. Jan Danser, Ewout J. Hoorn

**Affiliations:** 1 Divisions of Nephrology & Transplantation, Erasmus Medical Center, University Medical Center Rotterdam, Room Ns403, PO Box 2040, 3000 CA Rotterdam, The Netherlands; 2 Vascular Medicine and Pharmacology, Department of Internal Medicine, Erasmus Medical Center, University Medical Center Rotterdam, Rotterdam, The Netherlands; 3 Department of Internal Medicine, Leiden University Medical Center, Leiden, The Netherlands; 4 Section of Nephrology, Department of Internal Medicine, Amsterdam University Medical Centers, University of Amsterdam, Amsterdam, The Netherlands; 5 Division of Nephrology, Department of Internal Medicine, University Medical Center Groningen, University of Groningen, Groningen, The Netherlands

**Keywords:** Aldosterone, Clinical trial, Proteinuria, Renin

## Abstract

**Background:**

Acidosis-induced kidney injury is mediated by the intrarenal renin-angiotensin system, for which urinary renin is a potential marker. Therefore, we hypothesized that sodium bicarbonate supplementation reduces urinary renin excretion in patients with chronic kidney disease (CKD) and metabolic acidosis.

**Methods:**

Patients with CKD stage G4 and plasma bicarbonate 15–24 mmol/l were randomized to receive sodium bicarbonate (3 × 1000 mg/day, ~ 0.5 mEq/kg), sodium chloride (2 × 1,00 mg/day), or no treatment for 4 weeks (n = 15/arm). The effects on urinary renin excretion (primary outcome), other plasma and urine parameters of the renin-angiotensin system, endothelin-1, and proteinuria were analyzed.

**Results:**

Forty-five patients were included (62 ± 15 years, eGFR 21 ± 5 ml/min/1.73m^2^, plasma bicarbonate 21.7 ± 3.3 mmol/l). Sodium bicarbonate supplementation increased plasma bicarbonate (20.8 to 23.8 mmol/l) and reduced urinary ammonium excretion (15 to 8 mmol/day, both *P* < 0.05). Furthermore, a trend towards lower plasma aldosterone (291 to 204 ng/L, *P* = 0.07) and potassium (5.1 to 4.8 mmol/l, *P* = 0.06) was observed in patients receiving sodium bicarbonate. Sodium bicarbonate did not significantly change the urinary excretion of renin, angiotensinogen, aldosterone, endothelin-1, albumin, or α1-microglobulin. Sodium chloride supplementation reduced plasma renin (166 to 122 ng/L), and increased the urinary excretions of angiotensinogen, albumin, and α1-microglobulin (all *P* < 0.05).

**Conclusions:**

Despite correction of acidosis and reduction in urinary ammonium excretion, sodium bicarbonate supplementation did not improve urinary markers of the renin-angiotensin system, endothelin-1, or proteinuria. Possible explanations include bicarbonate dose, short treatment time, or the inability of urinary renin to reflect intrarenal renin-angiotensin system activity.

**Graphic abstract:**

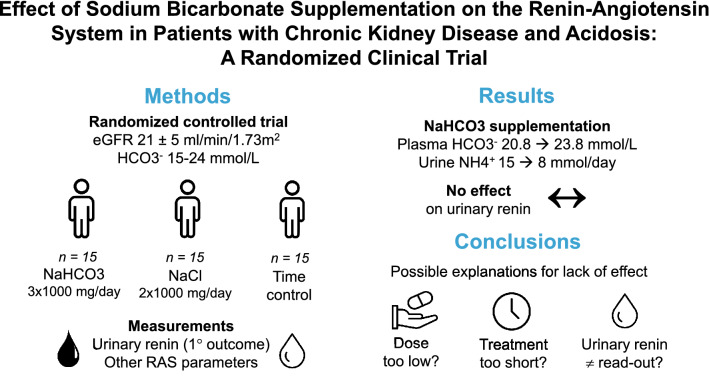

## Introduction

Metabolic acidosis is a common complication in patients with chronic kidney disease (CKD). The prevalence of metabolic acidosis (usually defined as a plasma bicarbonate concentration < 22 mmol/l) increases with higher CKD stage and is 26% and 47% for CKD stages G4 and G5, respectively [1,2]. Metabolic acidosis in CKD is associated with a more rapid progression of CKD [3–5]. A recent systematic review showed that metabolic acidosis is a modifiable risk factor for CKD progression as interventions with oral alkali supplementation or an alkaline diet reduce this risk [6]. However, the mechanisms of acidosis-induced kidney injury are incompletely understood.

Current understanding of how acidosis contributes to kidney injury suggests that acid retention triggers an adaptive response to increase ammoniagenesis [7]. This process is orchestrated by activation of the circulating and intrarenal renin-angiotensin systems (RAS) and endothelin-1. However, in a chronic setting and at the single-nephron level, this adaptive response may become maladaptive with the RAS and endothelin-1 contributing to inflammation and fibrosis [8]. For example, it has been shown that locally produced ammonium can activate the complement system with subsequent tubulo-interstitial inflammation and fibrosis [9]. In rats, induction of CKD with 2/3rd nephrectomy causes acid retention and higher levels of angiotensin II and aldosterone in the kidney; alkali treatment reverses these changes [10]. In clinical studies, alkali treatment reduced plasma and urinary aldosterone in patients with CKD stage G2 and G4 [11,12]. Similar findings have been reported for plasma and urinary endothelin-1 [10,13–16]. However, the activity of the intrarenal RAS is difficult to assess because it is questionable to what degree urinary RAS components truly reflect intrarenal RAS activity [17, 18].

Previous data suggest that the production of angiotensin II in the kidney depends on filtered (i.e., blood-derived) components of the RAS, including renin and angiotensinogen [19–21]. Accordingly, the modest alkali-induced lowering of urinary angiotensinogen in patients with CKD stage G3 could suggest reduced intrarenal angiotensin generation [22]. Alternatively, urinary angiotensinogen may simply follow the same urinary excretion pattern as albumin.^23^ Since this is not the case for urinary renin, [23, 24] this marker may be a more attractive parameter to assess intrarenal RAS activity. Accordingly, we hypothesized that sodium bicarbonate supplementation reduces urinary renin excretion in patients with CKD and metabolic acidosis. To address this, we performed an open-label clinical trial in which patients were randomized to receive sodium bicarbonate or sodium chloride, or served as time-controls. In addition to the measurement of plasma and urinary RAS parameters, we also analyzed the effects on urinary endothelin-1, albumin, α1-microglobulin, and complement.

## Methods

### Study design

We conducted an open-label randomized controlled trial at 4 study sites in The Netherlands, including Erasmus Medical Center, Rotterdam, Amsterdam University Medical Centers. University Medical Center Groningen and Leiden University Medical Center. The study was approved by the Medical Ethics Committee of the Erasmus Medical Center (MEC-2013–332). The trial was registered at clinicaltrials.gov with registration number NCT02896309. Patients were recruited from outpatient nephrology clinics between April 2014 and December 2018. All patients with CKD stage G4 (eGFR 15–30 ml/min/1.73m^2^) and with plasma bicarbonate levels between 15.0 and 24.0 mmol/l were eligible for inclusion. Exclusion criteria were sodium bicarbonate use in the month preceding the study, heart failure New York Heart Association class 3 or 4, liver cirrhosis with ascites and the inability to withdraw diuretics, systolic blood pressure > 140 mmHg despite the use of three different antihypertensive drugs, kidney transplantation, and use of calcineurin inhibitors. Patients were randomized for 4-week treatment with sodium bicarbonate (3 × 1000 mg/day, providing a sodium load of 36 mmol per day), sodium chloride (2 × 1000 mg/day, providing a sodium load of 34 mmol per day) or no treatment (time control). Allocation to treatment was done by randomization using sequentially numbered, opaque, sealed envelopes. Stratified randomization was used to ensure that a similar number of patients were allocated to each intervention at the different study sites.

### Measurements

At baseline and after 2 and 4 weeks, blood and 24-h urine samples were collected and office blood pressure was measured. Plasma and urine electrolytes, albumin, creatinine, and α1-microglobulin were measured at the Department of Clinical Chemistry of the Erasmus Medical Center. Venous blood gas analysis was performed directly after sample collection on a blood gas analyzer (ABL90 Flex Plus, Radiometer, The Netherlands; RAPIDLab 1265, Siemens, Germany). Estimated glomerular filtration rate (eGFR) was calculated using the CKD-EPI equation [25]. Creatinine clearance was calculated based on plasma and urinary creatinine excretion. Urinary ammonium was measured using the Berthelot-method, as described previously [26]. Plasma renin was measured using a radioimmunometric assay (Cisbio, Saclay, France). Urinary renin was measured using an in-house enzyme-kinetic assay that quantifies angiotensin I generation in the presence of excess sheep angiotensinogen [27]. In order to convert angiotensin I-generating activity to renin concentration, a conversion factor was used based on the fact that 1 ng Ang I/mL per hour corresponds to 2.6 pg renin/mL. Urinary angiotensinogen was measured as the maximum quantity of Ang I that was generated during incubation with excess recombinant renin using the same in-house assay [27]. Plasma and urinary aldosterone were measured by radioimmunoassay (Demeditec, Kiel, Germany). Endothelin-1 was measured using a Quantikine enzyme-linked immunosorbent assay (ELISA; R&D systems, Minneapolis, USA). Urine soluble terminal complement complex sC5b-9 was measured by ELISA as previously described [28]. All urinary excretions were expressed as ratio with urine creatinine to correct for any incomplete collections, as reported previously [29].

### Statistics

Data are presented as frequencies (percentages), mean ± standard deviation and median with 10th–90th percentile, as appropriate. The primary outcome was the change in urinary renin-to-creatinine ratio. A power calculation based on previous data indicated that a minimum of 45 patients (15 per treatment arm) was required to show that sodium bicarbonate supplementation would reduce urinary renin excretion by 0.3 ng/L (α = 0.025, β = 0.8, standard error 0.26) [23]. Secondary outcomes included the urinary-to-creatinine ratios of angiotensinogen, endothelin-1, albumin and α1-microglobulin. An exploratory analysis was performed for the treatment effects on kidney function, blood pressure and plasma potassium. The omnibus K2 test was used to test for normality. Non-normally distributed data were log-transformed for statistical analysis. Primary and secondary outcomes were analyzed using mixed linear models that included treatment and period (time) as fixed effects. In case a significant interaction between treatment and period was found, post-hoc tests were performed with correction for multiple comparisons according to Dunnett. Data were analyzed using SPSS Statistics (IBM, version 24.0). *P* < 0.05 was considered statistically significant.

## Results

### Baseline characteristics

Forty-seven patients entered the study protocol, of whom two patients discontinued treatment due to adverse reactions to sodium chloride supplementation (1 patient with gastrointestinal symptoms, 1 patient with polydipsia). Forty-five patients completed the study protocol (15 patients/arm). All patients that finished the treatment period were included in the analysis of the primary and secondary outcomes*.* The average age was 62 ± 15 years, 78% were males, the average eGFR was 21 ± 15 ml/min/1.73 m^2^, and the average plasma bicarbonate was 21.7 ± 3.3 mmol/l (Table 1).

**Table 1:** Baseline characteristics

	Total (*n* = 45)	NaHCO_3_ (*n* = 15)	NaCl (*n* = 15)	Time control (*n* = 15)	*P*-value
*Demographics*					
Age, years	62 ± 15	61 ± 17	61 ± 14	64 ± 14	0.9
Males, *n* (%)	35 (78)	11 (73)	14 (93)	10 (67)	0.2
European descent, *n* (%)	39 (87)	13 (87)	14 (93)	12 (80)	0.6
*Comorbidities*					
eGFR, mL/min/1.73m^2^	21 ± 5	21 ± 6	22 ± 3	20 ± 4	0.6
Systolic blood pressure, mmHg	137 ± 16	134 ± 10	126 ± 13	135 ± 22	0.3
Use of RAS-inhibitors, *n* (%)	39 (87)	13 (87)	12 (80)	14 (93)	0.6
Diabetes mellitus, *n* (%)	7 (16)	2 (13)	2 (13)	3 (20)	0.8
*Laboratory data*					
Plasma bicarbonate, mmol/l	21.7 ± 3.3	20.8 ± 3.9	21.8 ± 2.9	22.4 ± 3.1	0.4
Plasma potassium, mmol/l	5.0 ± 0.6	5.1 ± 0.7	5.0 ± 0.6	4.9 ± 0.5	0.6
Plasma renin, ng/l	41.8 (20.3, 119.4)	31.0 (18.4, 64.0)	75.2 (22.1, 130.4)	41.8 (16.0, 126.1)	0.5
Plasma aldosterone, ng/l	301 (175, 449)	236 (188, 427)	349 (155, 604)	332 (169, 415)	0.5
Urine sodium, mmol/mmol Cr	10.4 (8.8, 12.3)	10.4 (10.1, 13.7)	10.6 (8.5, 11.3)	9.8 (7.8. 13.5)	0.2
Urine ammonium, mmol/mmol Cr	1.2 (1.0, 1.6)	1.1 (0.9, 1.6)	1.0 (0.9, 1.9)	1.4 (1.1, 1.6)	0.6
Urine renin, ng/mmol Cr	1.1 (0.7, 2.0)	0.7 (0.5, 1.5)	1.1 (0.7, 2.1)	1.4 (1.0, 2.1)	0.5
Urine aldosterone, ng/mmol Cr	352 (272, 492)	337 (267, 632)	335 (289, 443)	353 (269, 461)	0.8
Urine angiotensinogen, µg/mmol Cr	3.4 (0.9, 14.5)	2.0 (0.6, 26.8)	7.1 (1.5, 12.3)	2.0 (1.2, 12.6)	0.9
Urine endothelin-1, ng/mmol Cr	0.02 (0.01, 0.05)	0.02 (0.01, 0.13)	0.02 (0.01, 0.02)	0.02 (0.02, 0.05)	0.3
Urine albumin, mg/mmol Cr	30 (5, 102)	30 (7, 93)	39 (6, 87)	16 (4, 96)	0.8
Urine α1-microglobulin, mg/mmol Cr	3.6 (2.0, 5.3)	3.9 (1.9, 6.4)	2.8 (2.2, 4.0)	3.8 (1.9, 6.2)	0.5

*Cr* creatinine, *eGFR* estimated glomerular filtration rate, *NaCl* sodium chloride, *NaHCO*_3_ sodium bicarbonate, *RAS* renin-angiotensin system

### Effects on acid–base status and the renin-angiotensin system

**Fig. 1:**
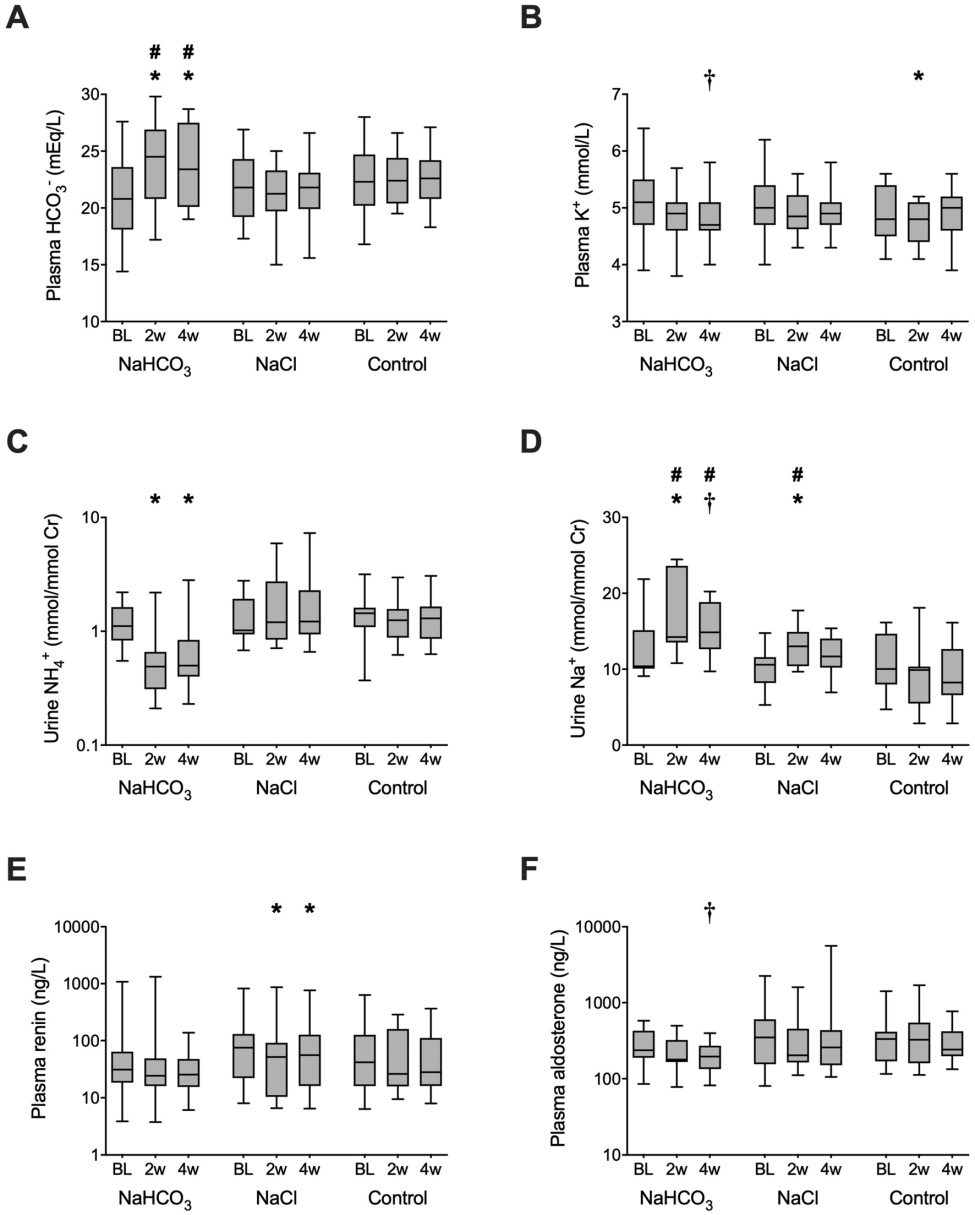
Sodium bicarbonate increased plasma bicarbonate and urinary sodium excretion and lowered urinary ammonium excretion, whereas sodium chloride treatment only increased urinary sodium excretion after 2 weeks of treatment. The horizontal black lines represent the median value; the lower and upper boundaries of the box represent the 25th and 75th percentiles; the whiskers represent the lowest and highest values. *HCO*_3_^−^ bicarbonate, *K*^+^ potassium, *Na*^+^, sodium, *NaCl* sodium chloride, *NH*_4_^+^ ammonium, *NaHCO*_3_ sodium bicarbonate. *P < 0.05 for the within-group difference from baseline. ^†^*P* = 0.07 for the within-group difference versus baseline; ^#^*P* < 0.05 for the difference versus control

Sodium bicarbonate supplementation increased plasma bicarbonate (with 3.0 ± 0.7 and 2.9 ± 0.8 mmol/L after 2 and 4 weeks of treatment, respectively; *P* < 0.01 *versus* baseline) and lowered urinary ammonium excretion (with − 7.0 ± 1.5 mmol/day and − 3.6 ± 1.9 mmol/day, *P* < 0.05 *versus* baseline, Fig. 1). No significant changes in plasma bicarbonate or urinary ammonium excretion occurred with sodium chloride supplementation and without treatment. Sodium chloride but not sodium bicarbonate supplementation significantly reduced plasma renin (with − 9.5 and − 7.9 ng/L, *P* < 0.05 *versus* baseline, Fig. 1). A trend towards a reduction in plasma aldosterone was observed with sodium bicarbonate supplementation after 4 weeks (− 99 ng/L, *P* = 0.07). No changes in the aldosterone-to-renin ratio were observed with either treatment (data not shown).

### Primary and secondary outcomes

In all three treatment groups, no significant within-group differences were detected in the urinary renin-to-creatinine ratio after two or four weeks of treatment (Table 2). In addition, no between-group differences were found. Similarly, sodium bicarbonate supplementation had no significant effect on any of the secondary outcome parameters (Table 2). In the within-group comparison, sodium chloride supplementation increased urinary angiotensinogen, albumin, and α1-microglobulin excretion; no between-group differences were shown for these outcomes (Table 2). Five patients did not use RAS-inhibitors. Two of these patients received sodium bicarbonate and this reduced urinary aldosterone (65% and 39% reduction after 2 and 4 weeks), an effect that was not observed with the other interventions. No effects on the other outcome parameters was observed. In addition, no differences were observed in a sensitivity analysis of the primary and secondary outcomes between patients with CKD stage G4a (eGFR 29–23 mL/min per 1.73 m^2^) and G4b (eGFR 22–16 mL/min per 1.73 m^2^). To determine whether correction of metabolic acidosis reduced the activity of the complement system, we also measured soluble terminal complement complex sC5b-9 in urine at the end of treatment. Urine sC5b-9 was undetectable in forty patients (< 0.05 U/ml). Of the five patients with detectable urinary complement, three had albuminuria > 1 g/day.

**Table 2:** Treatment effects on urinary renin-angiotensin, endothelin-1, and protein excretions

Measurement	Treatment	Baseline	2 weeks	4 weeks
Urine renin, ng/mmol Cr	NaHCO_3_	0.7, 0.4–2.2	0.9, 0.3–3.3	0.9, 0.2–3.1
	NaCl	1.1, 0.3–3.7	1.1, 0.1–4.3	1.2, 0.2–5.4
	Time control	1.4, 0.5–2.8	1.6, 0.5–2.4	1.8, 0.3–3.1
Urine aldosterone, ng/mmol Cr	NaHCO_3_	337, 171–1179	318, 125–513	356, 166–426
	NaCl	335, 142–787	**337, 126–627†**	317, 156–779
	Time control	353, 216–501	394, 142–676	293, 181–750
Urine angiotensinogen, µg/mmol Cr	NaHCO_3_	2.0, 0.4–83.5	4.3, 0.4–84.7	4.3, 0.2–104.5
	NaCl	7.1, 0.5–22.9	7.7, 0.4–30.9*	6.2, 0.4–39.0*
	Time control	2.0, 0.8–61.5	2.9, 0.9–55.3	5.5, 0.8–78.5
Urine endothelin-1, ng/mmol Cr	NaHCO_3_	0.02, 0.01–0.56	0.05, 0.01–0.31	0.02, 0.01–0.29
	NaCl	0.02, 0.01–0.07	0.02, 0.01–0.07	0.02, 0.01–0.07
	Time control	0.02, 0.01–0.06	0.02, 0.01–0.04	0.02, 0.01–0.05
Urine albumin, mg/mmol Cr	NaHCO_3_	30.4, 3.7–208.3	36.7, 5.8–196.9	40.4, 5.1–189.6
	NaCl	38.7, 1.6–124.4	46.4, 1.2–130.3*	58.6, 1.4–177.1*
	Time control	15.5, 1.9–171.2	17.6, 1.7–156.3	21.6, 2.1–160.7
Urine α1-microglobulin, mg/mmol Cr	NaHCO_3_	3.9, 1.4–10.5	5.6, 1.1–11.1	5.2, 1.3–11.1
	NaCl	2.8, 1.3–6.1	3.6, 1.3–8.0*	**3.4, 1.4–8.1†**
	Time control	3.8, 1.0–9.3	6.2, 1.5–9.5	4.5, 1.0–10.8

Statistically significant results are highlighted in bold

*Cr* creatinine, *NaCl* sodium chloride, *NaHCO*_3_ sodium bicarbonate

*P < 0.05 for the within-group difference versus baseline

^†^*P* = 0.06 for the within-group difference versus baseline

### Effects on kidney function, blood pressure and plasma potassium

Sodium bicarbonate or sodium chloride supplementation did not lead to significant changes in eGFR (Table 3). However, sodium bicarbonate did cause a small but statistically significant increase in urinary creatinine excretion after 4 weeks, which was not observed with sodium chloride treatment. No significant differences were identified for systolic and diastolic blood pressure within or between groups. After 4 weeks, there was a trend towards a reduction in plasma potassium with sodium bicarbonate (*P* = 0.06 for difference baseline *versus* 4 weeks), which was not observed with sodium chloride and without treatment.

**Table 3:** Effects of the sodium bicarbonate intervention on exploratory outcomes kidney function, blood pressure, and plasma potassium

Measurement	Treatment	Baseline	2 weeks	4 weeks
eGFR, mL/min/1.73m^2^	NaHCO_3_	21 ± 6	21 ± 5	21 ± 5
	NaCl	22 ± 3	22 ± 4	22 ± 4
	Time control	20 ± 4	20 ± 4	20 ± 4
Creatinine clearance, mL/min	NaHCO_3_	30 ± 10	29 ± 11	33 ± 12
	NaCl	39 ± 12	39 ± 14	39 ± 14
	Time control	32 ± 10	33 ± 10	30 ± 8
Creatinine excretion, mmol/day	NaHCO_3_	10.7, 8.7–13.4	10.2, 8.0–11.9	11.1, 10.3–14.0*
	NaCl	13.9, 10.9–17.0	12.5, 10.8–18.4	12.6, 10.7–16.6
	Time control	10.3, 8.1–17.7	12.9, 7.4–15.2	10.7, 8.0–15.1
Systolic blood pressure, mmHg	NaHCO_3_	134 ± 10	132 ± 18	132 ± 16
	NaCl	126 ± 12	125 ± 13	123 ± 13
	Time control	135 ± 22	140 ± 24	134 ± 20
Diastolic blood pressure, mmHg	NaHCO_3_	76 ± 10	75 ± 10	75 ± 10
	NaCl	78 ± 8	77 ± 9	78 ± 11
	Time control	81 ± 12	81 ± 10	78 ± 12
Plasma potassium, mmol/l	NaHCO_3_	5.1 ± 0.7	4.8 ± 0.5	**4.8 ± 0.5†**
	NaCl	5.0 ± 0.6	4.9 ± 0.4	4.9 ± 0.4
	Time control	4.9 ± 0.5	4.7 ± 0.4*	4.9 ± 0.5

Statistically significant results are highlighted in bold

*eGFR* estimated glomerular filtration rate, *NaCl* sodium chloride, *NaHCO*_3_ sodium bicarbonate

*P < 0.05 for the within-group difference from baseline

^†^*P* = 0.06 for the within-group difference from baseline

## Discussion

In this open-label, three-arm randomized controlled trial (RCT) we investigated whether sodium bicarbonate supplementation in patients with CKD and metabolic acidosis lowers urinary renin, as a potential measure of the intrarenal RAS. Sodium bicarbonate supplementation corrected metabolic acidosis and lowered urinary ammonium excretion. Despite these effects, we observed no within- or between-group differences for urinary renin. In addition, sodium bicarbonate had no significant effect on the urinary excretion of angiotensinogen, aldosterone, endothelin-1, albumin, or α1-microglobulin. Despite these negative findings, we believe our study adds three relevant aspects to the evolving field of metabolic acidosis in CKD.

First, several other clinical trials with sodium bicarbonate supplementation were also unable to show an effect on their primary endpoints. In this regard, our study is most comparable to the recent study by Raphael and colleagues who investigated the effect of sodium bicarbonate on urinary kidney injury markers [29]. In their placebo-controlled, double-blind RCT sodium bicarbonate was supplemented for six months at a dose of 0.5 mEq/kg to patients with type 1 or type 2 diabetes and an eGFR between 15 and 89 ml/min/1.73m^2^. Sodium bicarbonate supplementation increased plasma bicarbonate and reduced urinary ammonium, but did not reduce urinary TGF-*β*1, KIM-1, fibronectin, NGAL, or albumin. The most likely explanation for a lack of effect is that the dose of sodium bicarbonate was too low. Indeed, most RCTs that were unable to show an effect on the primary endpoint used a dose of 0.3–0.5 mEq/kg [29−31], whereas positive RCTs used a higher dose of approximately 1.0 mEq/kg [11,16,32]. To address this issue, Raphael et al*.* recently published a dose-finding study confirming that a higher dose of sodium bicarbonate (0.8 mEq/kg) had a stronger effect on plasma bicarbonate and urinary ammonium compared with a lower dose (0.5 mEq/kg). Wesson et al. showed that 0.5 mEq/kg sodium bicarbonate supplementation for 30 days did reduce plasma aldosterone and endothelin-1 levels in patients with CKD stage G1 or G2.[12] In agreement, we also observed that sodium bicarbonate reduced plasma aldosterone, although this was of borderline significance. The effect of sodium bicarbonate on aldosterone may be mediated by lowering of plasma potassium, although this was also of borderline significance in our study. In the RCT by Melamed et al. sodium bicarbonate supplementation also increased plasma bicarbonate and reduced plasma potassium [30]. In contrast, three previous studies did find effects of a lower sodium bicarbonate dose (0.3 or 0.5 mEq/kg) on urinary aldosterone, endothelin-1, angiotensinogen, albumin, and NAG, although the effect sizes were modest [12,15,16]. Possible explanations for the discrepancy with our study is that previous studies applied a longer treatment time (up to five years) and included patients with earlier stages of CKD. Finally, the use of RAS-inhibitors (used by 88% of the patients in this study) may suppress the RAS to an extent that alkali has no further effect.

A second issue that is raised by our study is whether urinary renin can truly be considered a marker of the intrarenal RAS. Determinants of urinary renin excretion include glomerular filtration, proximal tubular reabsorption, local production in the collecting duct, and intratubular conversion of plasma-derived prorenin to renin. In a study including 101 patients with or without diabetes mellitus and hypertension, urinary renin did not correlate with plasma renin and especially dissociated in patients with diabetes mellitus or on RAS-inhibitors. Accordingly, we proposed urinary renin to be a marker for the intrarenal RAS [33,34]. In a subsequent study, however, we showed that the glomerular sieving coefficient for renin is higher than for albumin and that variation in proximal tubular reabsorption explains the different urinary excretion patterns of renin and albumin [34]. We also showed that urinary renin does not reflect converted prorenin. A recent study in mice and humans with diabetes confirmed these concepts and did not find evidence for local production of renin [35]. Together these recent insights suggest that urinary renin excretion is mainly determined by variation in glomerular filtration and proximal tubular reabsorption and is therefore not a good marker for the intrarenal RAS. It would be of interest to explore whether renin mRNA or protein in urinary extracellular vesicles – which are mainly derived from tubular epithelial cells – is a better read-out of intrarenal RAS [36]. This also implies that positive effects of oral alkali may have been obscured by counteracting effects of the sodium load on filtration or reabsorption. We recently showed that an acid load increases albuminuria [37]. Therefore, correction of acidosis would be expected to reduce albuminuria, unless this effect is counterbalanced, for example by the sodium load. This could also explain why fruits and vegetables have more positive effects than sodium bicarbonate [38]. In this regard it would be interesting to assess the effect of oral alkali given with another cation. A clinical trial that compares the effects of potassium citrate with potassium chloride and placebo on kidney outcomes in CKD stage G3b and G4 is currently ongoing and may provide more insight into this matter [39.]

A third relevant finding in our study is that sodium chloride but not sodium bicarbonate increased albuminuria. This is relevant, because the dose-finding study by Raphael et al*.* observed an increase in albuminuria with the high dose (i.e., 0.8 mEq/kg per day) but not with the low dose (i.e., 0.5 mEq/kg per day) sodium bicarbonate [40]. The effect of alkali treatment on albuminuria is most likely the result of hemodynamic changes due to the sodium load given with bicarbonate. Another possibility is that a higher urine pH resulted in the detection of more intact albumin in the assay [41]. However, the results in our study and previous studies showing that sodium bicarbonate in both high and low doses also *lowers* albuminuria [11,15,22] suggest that assay characteristics do not fully explain the reported changes in albuminuria.

This is the first study to analyze the effect of sodium bicarbonate on urinary renin excretion. Another strength of this study is the inclusion of two control groups. However, this study also has a number of limitations. As discussed above, the dose of sodium bicarbonate or treatment time may explain why previously observed effects on aldosterone, endothelin-1, and proteinuria were not observed in this study. Although sample size was also modest, we recently showed in a study with a similar sample size that an acute acid load caused significant differences in urinary renin excretion between healthy subjects and patients with CKD [37]. Again, these results suggested that glomerular hyperfiltration or reduced proximal tubular reabsorption caused these changes in urinary renin. Therefore, the lack of effect of sodium bicarbonate supplementation on urinary renin likely means that no net changes in filtration or reabsorption occurred.

In conclusion, despite correction of acidosis and reduction in urinary ammonium excretion, sodium bicarbonate supplementation did not improve urinary markers of the renin-angiotensin system, endothelin-1, or proteinuria. Explanations for the lack of effect include bicarbonate dose, treatment time, or the inability of urinary renin to reflect intrarenal renin-angiotensin system activity.
